# Impact of climate change and infectious diseases: Implications for healthcare providers in the UK

**DOI:** 10.1016/j.fhj.2025.100239

**Published:** 2025-03-31

**Authors:** Christina Petridou, Amy Belfield

**Affiliations:** a The Rare and Imported Pathogens Laboratory UKHSA, Porton Down, Wiltshire, United Kingdom; b Hampshire Hospitals, NHS Foundation Trust, Hampshire, United Kingdom

**Keywords:** Infectious diseases, Climate change, Infections

## Abstract

Rising temperatures and changes in precipitation and humidity may affect the geographic ranges and habitats of pathogens and their animal hosts, and directly influence the reproduction, replication and transmissibility of certain pathogens and their vectors. These changes can lead to novel diseases presenting in new places, and a change in the seasonality of vector-borne diseases such as malaria, dengue and Lyme disease. Here we discuss the changing epidemiology of vector-borne diseases in the UK and abroad, and give some case examples. We will also discuss the importance of, and how to take, a detailed travel history and the impact of climate change on travel-associated infections.

The widespread effect of climate change on human and natural systems has led global health leaders to recognise climate change as the greatest health challenge of the 21st century.^1^ Warming surface and water temperatures, and the increase of extreme weather events such as flooding, have impacted ecosystems, infrastructure, livelihoods, food security and access to safe water resources, with poorer populations being disproportionately affected. Climate change also affects human health in many ways, including through its impact on infectious diseases. Changes in temperature, humidity and precipitation may affect the geographic ranges and habitats of pathogens and their animal hosts and directly influence the reproduction, replication and transmissibility of certain pathogens and their vectors. These changes can lead to novel diseases presenting in new places, and a change in the seasonality of vector-borne diseases.

Here we discuss the importance of, and how to take, a detailed travel history and the impact of climate change on travel-associated infections. We will also cover the changing epidemiology of vector-borne diseases in the UK and abroad and give some case examples.

## Changing epidemiology of climate sensitive diseases

Vector-borne diseases are viral, bacterial and parasitic infections transmitted to humans by infected mosquitoes, sandflies, ticks, tsetse flies and other vectors. They are a major global threat, with 80% of the world’s population at risk of one or more vector-borne disease^2^^,^^3^ (Box 1). Climate can affect the transmission dynamics, geographic spread and re-emergence of vector-borne diseases through direct effects on the pathogen, vector and hosts, and indirectly through its effect on ecosystem habitats.^4^

Box 1 **Importance of vector-borne diseases**

Alt-text: Box 1

**Table:** 

**Risk** 80% of the world’s population is at risk of one or more vector- borne disease.	**Mortality** and m**orbidity** Over 700,000 deaths are caused by vector-borne diseases annually. Many vector-borne diseases, such as onchocerciasis and filariasis, cause significant debilitation and suffering.	**Burden** 17% of the global burden of communicable diseases is due to vector-borne diseases, with poorer populations disproportionately affected. Low-income countries are most heavily impacted by climate change despite contributing least to greenhouse gas emissions.	**Major** e**xamples** Malaria, dengue, schistosomiasis, leishmaniasis, Chagas disease.

The most important climate-sensitive disease to mention is malaria, which is caused by infection with the protozoan parasite of the genus *Plasmodium*, transmitted by the bite of the *Anopheles* mosquito. Changes in temperature, rainfall and humidity due to global warming may influence malaria transmission and burden of disease both directly and indirectly.^5^ Examples include the expansion of malaria to the African highlands due to rising temperatures, areas that were previously on the fringes of endemic transmission.^5^ The increasing frequency of extreme weather events due to climate change such as such as flooding may also increase malaria numbers, as was shown in 2022 and 2023 in Pakistan where cases of malaria soared following extreme monsoon rainfall.^5^

During the 20th century, malaria was eradicated from many temperate areas, including Europe and the USA; however, mosquitoes that can transmit malaria are still present there. There is concern that, with climate change coupled with an increase in malaria importation events from endemic regions, there is the potential for malaria to reappear in countries where it was previously eradicated. The climate in southern Europe is still suitable for malaria transmission, and the increase in importation events means that localised outbreaks such as an outbreak of *P. vivax* in Greece in 2011 may become more frequent.^6^ In 2023, a year that saw a substantial increase in the number of imported malaria cases in the USA, autochthonous cases of malaria were reported in several US states for the first time in 20 years.^7^

Dengue is another important vector-borne disease to discuss; it is caused by four serologically related viruses transmitted by the *Aedes* mosquito. Over the past two decades, there has been a tenfold increase in reported cases, with 2024 being the worst year for dengue cases on record, driven by a huge upsurge in cases in the region of the Americas, with over 14 million cases reported globally.^8^^,^^9^ Rising temperatures have been linked to dengue becoming increasingly prevalent at higher altitudes, for example in Kathmandu, with Nepal recently experiencing its worst ever outbreak of dengue.^10^ *Aedes albopictus* is increasingly becoming established in large parts of Europe following its initial detection in Albania in the 1970s and *Aedes aegypti* is present in Cyprus, Madeira and the eastern shores of the Black Sea. The importation of dengue by viraemic travellers into these areas risks onwards transmission of the infection. Sporadic events of dengue virus transmission in Europe have been documented since 2010; however, over the past few years cases are becoming much more frequent, with 213 cases of autochthonous dengue reported in Italy and 83 in France in 2024.^11^ *Aedes* mosquitoes are also able to transmit Zika virus and chikungunya virus, and autochthonous cases of both infections have occurred in Europe.

Another important climate-sensitive pathogen is West Nile virus (WNV), which is spread from birds to humans via mosquitoes and can cause severe encephalitis and meningitis, especially in immunosuppressed people and those aged over 70 years. Within Europe, WNV has become endemic with an increase in the frequency, intensity and geographic expansion of outbreaks driven by more suitable climatic conditions.^12^ Higher temperatures have a direct effect on the incubation of WNV within mosquitoes, can accelerate mosquito development and biting rates. All cases of WNV in the UK have been acquired through travel. The bird hosts and *Culex* and other mosquito vectors are present in the UK, but the cooler temperatures have prevented the spread of WNV to northern European countries and to the UK. As the geographic spread of WNV expands further northwards with rising temperatures, the risk of viraemic migrating birds entering the UK may increase and in future years the potential of local transmission of WNV in the UK may be possible.

So far, we have focused on climate-sensitive mosquito-borne infections; however, warming temperatures affect the distribution and incidence of many other infections. Rising sea temperatures, for example, have led to more of Europe’s coastline becoming ecologically suitable for *Vibrio* species, putting more people at risk of severe skin and gastrointestinal infections, and previously unsuitable areas in central Europe are predicted to become suitable for the transmission of leishmaniasis.^12^ There is also evidence highlighting a rising trend in tick climatic suitability, amplifying the exposure to feeding ticks and the risk of tick-borne diseases such as Lyme disease and tick-borne encephalitis (TBE); a good example of this is the emergence of Lyme disease in Canada.^13^ *Ixodes ricinus*, the tick vector for TBE, is widely established across the UK. Over the past 5 years, there have been several cases of UK-acquired TBE, a disease previously only detected in returning travellers from endemic areas. Since 2019, tickborne encephalitis virus (TBEV) has been detected in ticks in several areas of the UK, including Thetford Forest and North Yorkshire. Box 2 describes the first case of TBE likely acquired in the UK.

Box 2 Alt-text: Box 2

**Table:** 

In 2019, a 3-month-old infant developed fevers and seizures 11 days after being bitten by a tick in the New Forest area of England. The child had a lymphocytic CSF and TBE IgM and IgG were positive, which was highly suggestive of TBE. This was the first highly probable case in the UK.^14^ Since then, there has been one further case of probable TBE diagnosed serologically and two cases confirmed by polymerase chain reaction.^15^
Learning points: Emerging infections may become established in places where the disease was not previously found, for example through migratory birds or animal movement, and clinicians need to be aware of this. TBE is a clear example of this, as incursion events likely happened and now it is present in the UK tick population. Clinicians now need to consider the diagnosis of TBE not only in travellers from endemic areas, but also in non-travellers.

## Travel-associated infections in the UK

**Fig. 1:**
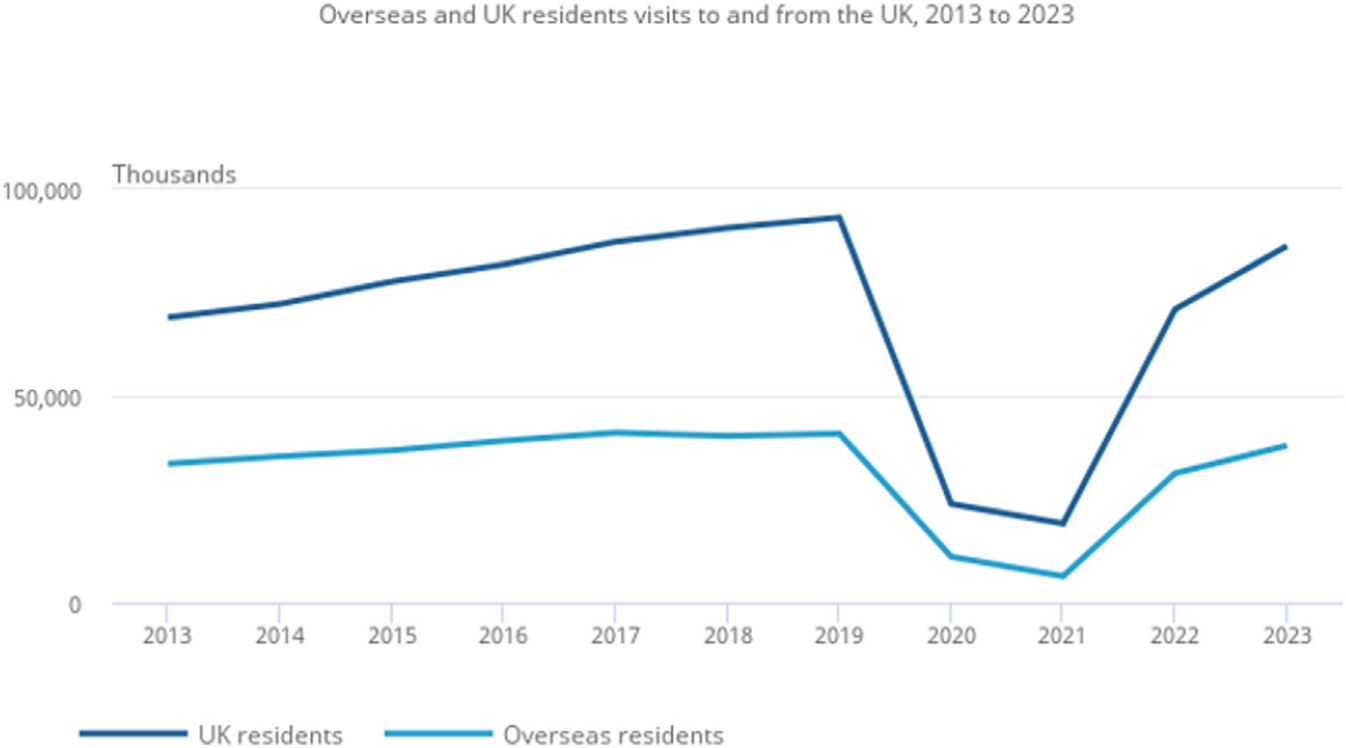
Visits to and from the UK taken from ONS (2024). Travel trends: 2023 [online] gov.uk ONS Available at: https://www.ons.gov.uk/peoplepopulationandcommunity/leisureandtourism/articles/traveltrends/2023.

The easing of COVID-19-related travel restrictions in 2021 saw an increase in global travel. In 2022, UK residents made 71 million visits abroad, a fourfold increase compared to the previous year (with a further increase in 2023 to 8.6 million visits (Fig. 1)).^16^

Mediterranean countries are the most frequently visited countries by UK residents. In 2023, 21% of UK residents visited Spain, 11% visited France and 6% visited Italy, a pattern also seen in previous years.^16^ Worldwide, an estimated 1.1 billion international tourist arrivals were recorded from January to September 2024, with most destinations exceeding pre-pandemic levels.^17^

This increase in travel, along with an increase in cases of vector-borne and other climate-sensitive infections worldwide, means that there will be a higher likelihood of patients presenting to hospital and general practice with travel-associated infections. Clinicians need to recognise the importance of taking a travel history to ensure that important infections, such as malaria, are not missed. Whether a patient has travelled or not should form part of the initial screening process in emergency departments, not only to inform the differential diagnosis and identify patients at risk of rapid deterioration, but also to recognise patients with a potential transmissible infection that may have significant public health implications.

**Fig. 2:**
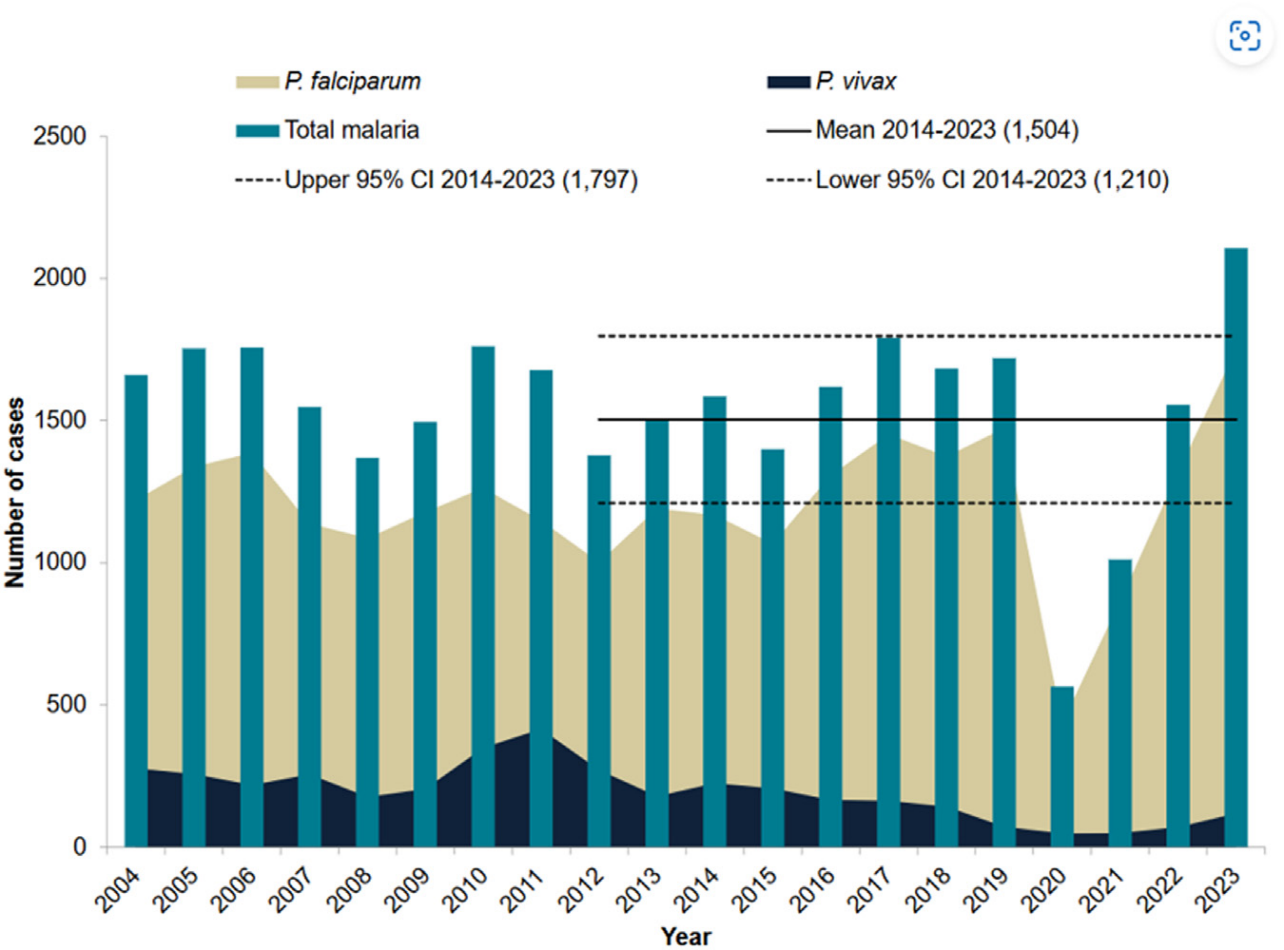
Cases of malaria in the UK: 2004 to 2023 taking from the following: UKHSA (2024). *Malaria imported into the UK: 2023*. [online] gov.uk UKHSA Available at: https://www.gov.uk/government/publications/malaria-in-the-uk-annual-report/malaria-imported-into-the-uk-2023.

The estimated number of global malaria cases in 2022 exceeded pre-COVID-19 pandemic levels in 2019.^5^ This increase in cases, coupled with an increase in the number of travellers arriving in the UK, has meant that the number of malaria cases diagnosed in the UK also significantly increased in 2023 (Fig. 2). Malaria caused by *Plasmodium falciparum* accounts for the greatest number of cases, reflecting the global epidemiology of the disease, and most infections occur in people visiting family and friends in West Africa.^18^ Worryingly, initial treatment failures due to drug resistance represent a tiny but increasing proportion of *P. falciparum* cases in the UK.^18^ Box 3 gives a real-life example of a missed infection, which may have had disastrous consequences.

Box 3 Alt-text: Box 3

**Table:** 

A 32-year-old normally well Cameroonian woman presented to the emergency department of her local district general hospital on 21 September 2024 complaining of a fever, vomiting and body aches for the past 48 h. She was reviewed by the registrar and diagnosed as having a ‘viral infection’ and sent home. There was no documentation of a travel history being taken. The patient then represented to a neighbouring emergency department 2 days later complaining of ongoing fevers and that she was feeling more unwell. This time a travel history was taken, and it transpired that she had returned from Cameroon on 16 September 2024. She had been visiting her family and had not been taking malaria prophylaxis. She was diagnosed with *Plasmodium falciparum* malaria and had a parasitaemia of 1%. She was given malaria treatment and made an uncomplicated recovery.
Learning points: Patients will not always volunteer a travel history; this can be for many reasons, including them not realising the importance of it, so the question must be asked. Malaria is the most common life-threatening tropical infection in returning travellers who present with a fever.

2024 saw the highest number of imported dengue cases into the UK since surveillance commenced in 2009, with most cases in travellers from Barbados and Brazil, reflecting the global picture.^19^ Clinicians need to be aware of the risk of dengue, chikungunya and Zika viruses, not only from people returning from the tropics but also increasingly from Europe. *Aedes albopictus* is an invasive mosquito species and well adapted to urban environments and can overwinter. Currently it is not established in the UK; however, since 2016 there have been multiple incursions detected in England, which may become more frequent as the mosquito becomes more widespread in Europe. Currently, endemic transmission of dengue is limited by climatic variables; however, this may change in years to come due to climate change.^20^ Parts of south-east England are already suitable for *Aedes albopictus*, and climate modelling under a high emissions scenario suggests that *Aedes albopictus* has the potential to become established in most of England and southern Wales by the 2060s, meaning that parts of the UK would be at risk of endemic transmission of infections such as dengue.^21^ Vector surveillance in mosquito importation risk areas such as along motorways is important to prevent vector establishment.

## How to take a travel history

To narrow the differential diagnosis in unwell travellers, a thorough history needs to be taken. Firstly, the geographic area of travel needs to be properly identified in detail. Identifying a broad area of travel is inadequate, as the profile of illness may vary greatly within a region or even within a country. Examples of this include Lyme disease, which is endemic in the USA; no cases were reported in Oklahoma state in 2022, compared to 16,798 in New York State alone.^22^ Another important example is Lassa fever, which is endemic in Nigeria; each state had suspected cases in 2024, but Zamfara state only had three suspected cases compared to Ondo, which had 2,613.^23^ In addition, infections have different incubations periods, so defining the timing of exposures for different geographic areas is important and documenting travel dates is paramount. This may help exclude some infections immediately from the diagnosis. Most infections will present within a month of travel; however many infections, such as hepatitis E, rabies and *Plasmodium vivax*, may present later.

Being thorough and asking about exposures and travel-related activities such as freshwater exposure, sexual activity and animal and insect exposure can help narrow down the differential significantly. Table 1 gives an outline of the important questions to ask when taking a travel history and explains why.

It is very important to remember that many illnesses in returning travellers are due to common and cosmopolitan infections such as influenza and pneumococcal pneumonia, which must always be thought of in addition to the more tropical and rarer infections. It is also necessary to consider non-infectious causes in unwell travellers, such as the development of a deep vein thrombosis or pulmonary embolism due to long-haul travel.

When reviewing returning travellers, one of the immediate considerations should be whether the patient needs isolation due to a transmissible infection. The most important example of this would be a high-consequence infectious disease (HCID) such as Lassa fever or Middle East respiratory syndrome (MERS) and transmission within healthcare settings of these infections is known to occur. Healthcare workers are at particular risk of infection if appropriate infection prevention and control practices are not followed. Other examples of transmissible infections to consider in returning travellers include measles and meningococcal meningitis; Box 4 shows a real-life example the clinical impact when infection prevention and control practices are not adhered to. Apart from patients and clinical staff who may be at high risk, it is important to not forget laboratory staff who may be handling highly infectious samples.

Box 4 Alt-text: Box 4

**Table:** 

A 42-year-old man presented to the local rmergency department with a temperature of 39.6°C, a sore throat and a widespread maculopapular rash. He was hypoxic with oxygen saturations of 86% on air. He had returned from Bucharest 2 days before, where he had been visiting his family. His English was very poor, and a thorough travel history and vaccination history were not taken initially. The infection team were contacted early to discuss the case; however, they were not informed that the patient had a widespread rash, and the patient was treated for an atypical pneumonia. A CTPA showed evidence of pneumonitis. Measles was not considered until 4 days after the patient was admitted, and a viral swab was collected and was measles PCR positive; the patient had never been vaccinated against measles. The patient had not been isolated immediately and airborne precautions had not been used, leading to a huge amount of contact tracing and significant number of staff and patient contacts in several different wards and departments.
Learning points: Never forget that you might be managing a patient with a highly transmissible disease and, when reviewing a patient, always consider whether they should be isolated and what precautions need to be used. Notify your infection department early and make sure that you have a full history, and ensure the infection prevention and control team are contacted early.

**Table 1:** Questions to ask when taking a travel history and why.

Question	Reasoning
Where did they travel? (Get specifics of areas visited; place names, rural/urban, any layovers?)	Different areas, even within a country, have different disease prevalence.
When did they travel? And when did they return to the UK?	Longer stays increases risk of exposure.
When did they become unwell?	Different infections have different incubation periods. If within 21 days of travel to an endemic area, viral haemorrhagic fevers (VHFs) must be ruled out.^a^
Pre-travel advice?	Pre-travel vaccinations may help rule out some infections, but also can cause cross-reactivity with some serology tests. Malaria prophylaxis reduces (but does not eliminate) the chances of getting malaria.
What type of accommodation? Rural or urban?	Increased risk of VHF eg Lassa fever if rural or poor living standards and increased risk exposure to vectors.
Food or drink?	Tap water? risk of GI infections eg giardiasis, typhoid, cholera. Unpasteurised milk? risk of brucellosis, Q fever, tuberculosis etc. Bush meat? risk of VHF eg Ebola^a^. Street vendors? risk of *Campylobacter, Salmonella*, hepatitis A etc.
Freshwater exposure?	Increases risk of certain infections eg leptospirosis, schistosomiasis, amoebiasis
Sexual contacts?	Increased risk of HIV, hepatitis B/C, Mpox, chlamydia and gonorrhoea (may be more resistant to antibiotics)
Any unwell contacts?	If similar symptoms, may help narrow differential.
Any hospital visits or medical care abroad?	Increases risk of antimicrobial drug resistance and VHF.^a^
Any animal contacts?	Increases risk of infections such as hepatitis A/E, *Salmonella* and *Campylobacter*. May also increase the risk of VHFs.^a^
Any animal bites?	Risk of rabies/tetanus and certain bacteria
Any tick bites?	Vectors for certain diseases eg tick-borne encephalitis, rickettsial diseases and Crimean-Congo haemorrhagic fever^a^

a In endemic areas where VHFs are present. See ACDP Viral Haemorrhagic Fevers Risk Assessment (October 2024) for complete assessment vhf-risk-assessment-algorithm-23-october-2024.pdf.^24^

As previously mentioned, involving your local infection team early is very important to ensure that appropriate tests are done quickly and the correct treatment is commenced. When discussing with your infection team, ensure that you have a full and thorough history (Table 1) ready to avoid any delays or missed opportunities. Discussions may then need to be had with specialist reference laboratories and clinicians, such as the Malaria Reference Laboratory or the Imported Fever Service, to advise on specific diagnostics and treatment; however, these discussions would generally happen with one of the infection team.

## Closing remarks

The warming climate is expected to increase the risk of several vector-, food- and water-borne infections as discussed; however, it is important to note that the relationship is complex and multifactorial. Changes to vector habitats such as rapid urbanisation bring people into more frequent contact with competent vectors such as *Aedes*, increasing the risk of explosive outbreaks. Population displacement due to extreme weather events, and the effect on agriculture and food insecurity, all affect the risks of people developing infections. Indirect effects of climate change on health may also happen through reduced access to medicines and commodities such as insecticide-treated nets. The risk of future epidemics and outbreaks depends not only on changes in the climate, but crucially on access to clean water, sanitation, economic stability and access to healthcare. Changes in land use and socioeconomic factors will have a huge impact on whether infectious diseases will become established. The trajectory of infections in the future will depend on investment in surveillance and outbreak preparedness, and prevention and control interventions.

### Learning points

• Malaria is a serious and life-threatening condition and is the most important travel-related infection to never miss. Patients returning from tropical regions with a fever should be taken seriously and malaria excluded – despite effective treatments and chemoprophylaxis being available, patients in the UK die every year from malaria.
• Many mosquito-borne diseases are becoming more frequent, and infections that were previously thought to only occur in tropical and subtropical areas such as dengue are now detected in southern Europe, in countries most frequented by UK tourists, on a regular basis. Doctors need to be aware of this when reviewing returning travellers.
• Global travel has increased to pre-pandemic levels, increasing the chances of clinicians coming across returning ill travellers. Doctors need to understand the importance of taking a good travel history.

## Funding

This research did not receive any specific grant from funding agencies in the public, commercial, or not-for-profit sectors.

## CRediT authorship contribution statement

**Christina Petridou:** Writing – review & editing, Writing – original draft, Resources. **Amy Belfield:** Writing – review & editing, Resources.

## Declaration of competing interest

The authors declare that they have no known competing financial interests or personal relationships that could have appeared to influence the work reported in this paper.
